# Modelling Selective CO_2_ Absorption and Validation via Photosynthetic Bacteria and Chemical Adsorbents for Methane Purification in Anaerobic Fermentation Bioreactors

**DOI:** 10.3390/ma16196533

**Published:** 2023-10-01

**Authors:** Yu-Chen Hsu, Shunnian Wu, Juei-Yu Chiu, Hashan N. Thenuwara, Hasanthi L. Senevirathna, Ping Wu

**Affiliations:** 1 Department of Environmental Science and Engineering, National Pingtung University of Science and Technology, Pingtung 91201, Taiwan; 2 Entropic Interface Group, Engineering Product Development, Singapore University of Technology and Design, 8 Somapah Road, Singapore 487372, Singaporehashan_thenuwara@mymail.sutd.edu.sg (H.N.T.);

**Keywords:** bulk and surface modelling, MgO-Mg(OH)_2_ composites, selective CO_2_ absorption, methane purification, photosynthetic bacteria (PNSB), anaerobic fermentation bioreactors

## Abstract

This study delves into advanced methane purification techniques within anaerobic fermentation bioreactors, focusing on selective CO_2_ absorption and comparing photosynthetic bacteria (PNSB) with chemical adsorbents. Our investigation demonstrates that MgO-Mg(OH)_2_ composites exhibit remarkable CO_2_ selectivity over CH_4_, substantiated through rigorous bulk and surface modelling analyses. To address the challenges posed by MgCO_3_ shell formation on MgO particles, hindering CO_2_ transport, we advocate for the utilisation of MgO-Mg(OH)_2_ composites. In on-site experiments, these composites, particularly saturated MgO-Mg(OH)_2_ solutions (S2), achieved an astonishing 100% CO_2_ removal rate within a single day while preserving CH_4_ content. In contrast, solid MgO powder (S3) retained a mere 5% of CH_4_ over a 10 h period. Although PNSB (S1) exhibited slower CO_2_ removal, it excelled in nutrient recovery from anaerobic effluent. We introduce a groundbreaking hybrid strategy that leverages S2’s swift CO_2_ removal and S1 PNSB’s nutrient recovery capabilities, potentially resulting in a drastic reduction in bioreactor processing time, from 10 days when employing S1 to just 1 day with the use of S2. This represents a remarkable efficiency improvement of 1000%. This pioneering strategy has the potential to revolutionise methane purification, enhancing both efficiency and sustainability. Importantly, it can be seamlessly integrated into existing bioreactors through an additional CO_2_ capture step, offering a promising solution for advancing biogas production and promoting sustainable waste treatment practices.

## 1. Introduction

As a naturally occurring and renewable energy source, biogas, which consists mainly of carbon dioxide–methane (CO_2_-CH_4_) mixtures, has emerged as an alternative fuel to natural gas. However, the presence of CO_2_ can reduce the heating value and generate greenhouse gases. Therefore, the effective separation of CO_2_ and CH_4_ in biogas streams through targeted CO_2_ reduction is critical for the practical application of biogas.

Various separation techniques have been developed to solve the problem, such as absorption, membrane separation, cryogenic separation, and adsorption [1]. While the photosynthetic bacteria system offers the advantage of simultaneous CO_2_ capture and methane content enhancement [2], it is essential to acknowledge that its economic viability can be compromised by challenges in cultivation techniques [3,4]. The current materials and methods are less cost- and time-effective and should be redesigned based on this research finding.

Adsorption is considered to be a competitive solution, and has the advantages of simple process, mild operating conditions, great operating flexibility, wide operating temperature range, low operating cost, stable performance, and no corrosion and fouling [4]. Currently, there are a number of experimental studies that provide good data for the adsorption of pure CO_2_ or pure CH_4_ on microporous materials, such as oxides [5,6], activated carbons [7,8], metal–organic framework materials [9,10], and zeolites [11,12]. However, the number of studies on the adsorption properties of the CO_2_/CH_4_ mixture is limited, although recent experimental studies show that selective adsorption of CO_2_ is possible [13,14,15,16,17,18]. The theoretical simulation of the separation of CO_2_ from CO_2_/CH_4_ mixtures by microporous MOFs by Bastin et al. [19], and adsorption behaviour of an equimolar CO_2_/CH_4_ mixture in carbon nanotubes (CNTs) by Huang et al. [20], show that the use of adsorption techniques is useful for the selective adsorption of CO_2_. The CNTs have the best selectivity for the binary CO_2_/CH_4_ mixture when the selectivity of common adsorbents like activated carbons, zeolites, MOFs, and others is compared [20]. The selective CO_2_ adsorption capability of the adsorbent is the vital component for their industrial application.

Due to its substantial theoretical CO_2_ capture capability (1100 mg CO_2_/g adsorbent), MgO-based composites have been identified as a promising CO_2_ absorbent. However, commercial MgO at 50 °C has a relatively low CO_2_ adsorption capacity of 8.8 mg/g [21], whereas porous MgO produced by the thermal breakdown of Mg(OH)_2_ has a 33 mg/g CO_2_ removal capacity [22]. MgO particles react with CO_2_ to create MgCO_3_, which surrounds the unreacted MgO particles and prevents CO_2_ molecule diffusion [6,23]. To overcome the carbonate blocking effect, we adopted water-harvesting strategies from a genus of Namib Desert beetles [24]. The Stenocara beetle’s back is covered in many hydrophilic bumps that are not waxy and are surrounded by a hydrophobic wax-coated background. The alternate hydrophobic and hydrophilic surface domains promote water generation and adsorption. Recent research [25] has found that well-structured combinations of strong CO_2_ adsorbents, like MgO, and weak CO_2_ adsorbents, like Mg(OH)_2_, can greatly increase the practical CO_2_ adsorption capacity. The adsorption of CH_4_ is anticipated to remain minimal due to the restricted interaction between CH_4_ and the surface OH- group on the Mg(OH)_2_ surface. The current study suggests using MgO-Mg(OH)_2_ composite as chemical absorbents for CO_2_ and CH_4_ separation in biogas, in light of these insights. While admitting the inherent constraints in CH_4_ adsorption brought on by the weaker interaction with the Mg(OH)_2_ surface, the interweaving of both materials offers the possibility of increased CO_2_ capture efficiency. Additionally, the use of CO_2_ by microalgae or anaerobic photosynthetic bacteria is a rapidly expanding technology for energy conservation. In order to upgrade the methane gas produced by a pig farm, we conducted our research in photobioreactors employing purple non-sulphur bacteria (PNSB) and composite materials made of MgO-Mg(OH)_2_ [26].

This study started by simulating the selective absorption of CO_2_ over CH_4_ by MgO-Mg(OH)_2_ composites using both bulk thermodynamic and surface Density Functional Theory (DFT)-based modelling. The results from the modelling establish the groundwork for the later experimental validations. For validation purposes, both biological and chemical experimental methodologies were used. First, biological PNSB was introduced as an absorption agent (S1), which is renowned for its cutting-edge capabilities in wastewater treatment and bioresource recovery. The second method involves chemical absorption utilising two substances: an aqueous solution of MgO-Mg(OH)_2_ (S2) and solid MgO powder (S3). On-site sampling was conducted at the anaerobic fermentation methane outlet of a pig manure solid–liquid separation-free bioreactor. Methane gas produced from the anaerobic fermentation of solid–liquid separated free livestock waste was passed through a desulphurization tower and subsequently injected into the bioreactor, allowing for a 10-day shaken culture experiment. To compare the adsorption effects of the biological and chemical techniques, variations in CO_2_ and CH_4_ were continually measured throughout the observation time. The findings of the experiments were compared to those predicted by the models, and both biological and chemical methods underwent careful analysis. In the end, a synergistic strategy combining PNSB (S1) and MgO-Mg(OH)_2_ aqueous solution (S2) is suggested.

## 2. Methodology

This study employs a combination of modelling prediction and experimental validation methods to investigate CO_2_ selectivity in the S1, S2, and S3 systems.

### 2.1. Theoretical Calculations

For computer modelling, two techniques are utilised: (1) bulk thermodynamic calculations using the commercial software FactSage (Centre for Research in Computational Thermochemistry, Montreal, Canada) [27] and (2) DFT calculations using the Vienna ab initio simulation package (VASP) (VASP Software GmbH, Vienna, Austria) [28] with the Perdew–Burke–Ernzerhof (PBE) generalised gradient approximation (GGA) exchange–correlation functional [29].

#### 2.1.1. Bulk Thermodynamic Calculations for CO_2_ and CH_4_ Absorption Using MgO and Mg(OH)_2_, Respectively

In our thermodynamic modelling, we utilised the Equilib module from FactSage [27] to compute the chemical equilibria involving CO_2_ (gas) and CH_4_ (gas) in conjunction with MgO and Mg(OH)_2_. The calculations incorporated thermodynamic data for all relevant compounds, as provided in the FactPS and FToxid databases. These calculations were conducted at a temperature of 25 °C and a pressure of 1 ATM.

#### 2.1.2. DFT Calculations of CO_2_ and CH_4_ Absorption on MgO and Mg(OH)_2_ Surfaces

In order to investigate the surface absorption of CO_2_/CH_4_ on MgO and Mg(OH)_2_, a projector augmented wave (PAW) method [30,31] was adopted as a plane-wave basis set to describe the electron–core interaction. The kinetic energy cutoff for the plane-wave expansion was set at 500 eV. The van der Waals contribution was taken into account using the DFT+D3 correction technique developed by Grimme et al. [32]. The total energy convergence was set as 1.0 × 10^−6^ eV, and the force on each individual atom was minimised to be smaller than 0.01 eV/Å for geometry optimisation and total energy calculations. The value for smearing was fixed to 0.01 eV. Monkhorst−Pack [33] K-points mesh was used for sampling the Brillouin zone, with the K-points number (N_K_) being adjusted to keep (N_K_ × L) and with L being the lattice constant equal to ~45 Å for structural relaxations and ~75 Å for electronic calculations, respectively.

The previously published [34] optimised MgO and Mg(OH)_2_ crystalline structures were used in this work. MgO and Mg(OH)_2_ were both cleaved in the most stable (001) orientation in order to examine their adsorption of CO_2_ and CH_4_ [35,36]. To make sure that the interaction force between the layer planes was sufficiently small, the vacuum between them was 20 Å thick. MgO slabs are composed of six layers of the 3×3 expansion of the MgO unit cell. The adsorbate molecule and top 3 layers were free to relax, while the bottom 3 layers remained fixed in their bulk placements. Mg(OH)_2_ slabs are composed of three layers of the 4×4 expansion of the Mg(OH)_2_ unit cell. The adsorbate molecule and top two layers were free to relax while the bottom layer was held in its bulk position.

The adsorption energy $E_{ad}$ of the adsorbate molecule X (X = CO_2_ or CH_4_) on the MgO and Mg(OH)_2_ surface is defined as $E_{ad}=E_{surface+X}-E_{X}-E_{surface}$, where $E_{surface+X}$ is the total energy of the surface and adsorbate molecule, $E_{X}$ is the energy of the adsorbate molecule CO_2_ or CH_4_, and $E_{surface}$ is the total energy of the surface. A lower value of $E_{ad}$ denotes the stronger molecule’s adsorption on the surface. The charge of an atom was defined as the difference between the valence charge and the Bader charge. The Bader charge was calculated using the Bader scheme of charge density decomposition [37,38].

### 2.2. Materials and Reagents

Chemicals with a purity of over 95% and 200 mL drip bottles with sealed caps were procured from Nihon Shiyaku Industries Ltd. (Osaka, Japan). The PNSB used in the study consisted of Rhodospirillum, Rhodopseudomonas, and Rhodomicrobium, which constitute the major microbial communities provided by the Food Research Institute. Rhodopseudomonas palustris makes up the majority of the bacterial communities among them. The components of the bacterial growth medium are detailed in Table 1.

In our earlier study, we reported on the synthesis and characterisation of MgO-Mg(OH)_2_ composites [25].

### 2.3. Separation Measurement of S1, S2, and S3 Systems, Respectively

Dry heat sterilisation was applied to a 200 cc drip bottle over the course of six hours in an oven set to 160 °C. Three different types of separation experiments were carried out in a SAN-C301 biological safety cabinet from San-Hsiung Technology Co., Ltd. (Kaohsiung City, Taiwan): (1) introducing S1, comprising 100 mL of PNSB liquid with a cell concentration of 106 cells/mL, into each of the five sterilised bottles or bioreactors, (2) introducing S2, consisting of 20 g of MgO powder and 100 mL of H_2_O, into each of the five bioreactors, and (3) adding S3, including 20 g of magnesium oxide only, into each of the five bioreactors, once the bioreactors have cooled down. Three distinct types of bottles were agitated at 150 rpm, serving as photobioreactors for biogas purification. Subsequently, each of the five duplicate bottles was filled with gas via the methane vent from the Central Livestock Farm. Following this, each bottle was promptly sealed using a rubber stopper and secured with an aluminium cover. These bioreactors were then placed within a growth chamber set to maintain a temperature of 25 °C, operating under a 16/8 h light/dark cycle with a light intensity of 3000 lux.

To determine the concentrations of CO_2_ and CH_4_, we employed Shimadzu GC-8A GC-TCD (Shimadzu Scientific Instruments (Taiwan) Co., Ltd., Taipei City, Taiwan) equipped with a Shimadzu SUS column (4 × 3.0 × 3.0 m) packed with Porapax Q50/80 mesh material. The injection temperature, detector temperature, and oven temperature were set at 150 °C, with the oven temperature held at 45 °C. Helium served as the carrier gas. The concentration of CH_4_ and CO_2_ was determined using a calibration curve established via a standard gas mixture (55% CH_4_, 20% CO_2_, and 25% He) obtained from Jing De Gases Co., Ltd. (Kaohsiung City, Taiwan)

## 3. Results and Discussion

### 3.1. Bulk Thermodynamic Calculations

Table 2 calculates and summarises the chemical reactions for equilibrium CO_2_ and CH_4_ absorption in S2 and S3 systems. The calculated products, which might not be achieved because of unfavourable kinetics, are thermodynamic equilibrium products.

As can be observed from Table 2, when MgO reaches the equilibrium reaction with H_2_O, it can completely transform into Mg(OH)_2_, as shown in Equation (1). Equations (2) and (3) demonstrate that an equal amount of MgCO_3_ was created by MgO and Mg(OH)_2_ with a 100% reaction consuming the same amount of CO_2_. However, neither MgO nor Mg(OH)_2_ are anticipated to react with CH_4_. Even though Equations (2) and (3) predict that MgO and Mg(OH)_2_ can absorb 100% of the CO_2_, kinetic restrictions may prevent their implementation. For instance, the production of MgCO_3_ shells around the core MgO particles greatly slows down the CO_2_ absorption process [6]. Contrarily, physical adsorption attributed to structural advantages or electrostatic interaction may have a considerable impact, even though Equations (4) and (5) predict 0% CH_4_ absorption by MgO and Mg(OH)_2_. The following in-depth analysis of the surface absorption of CO_2_/CH_4_ on MgO and Mg(OH)_2_ surfaces is therefore required.

### 3.2. Surface DFT Calculations

**Figure 1:**
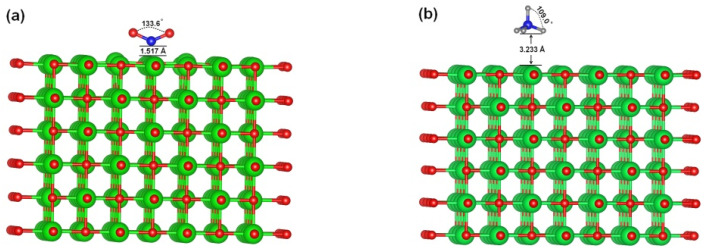

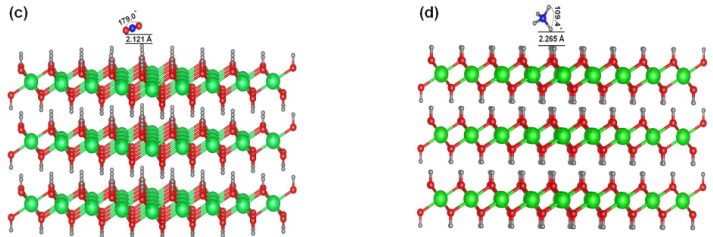
The optimised configuration of CO_2_ or CH_4_ adsorption on MgO or Mg(OH)_2_ surface, respectively. (**a**) CO_2_ on the MgO surface, (**b**) CH_4_ on the MgO surface, (**c**) CO_2_ on Mg(OH)_2_ surface, and (**d**) CH_4_ on Mg(OH)_2_ surface. Green, red, blue and grey balls represent Mg, O, C and H atoms, respectively.

**Figure 2:**
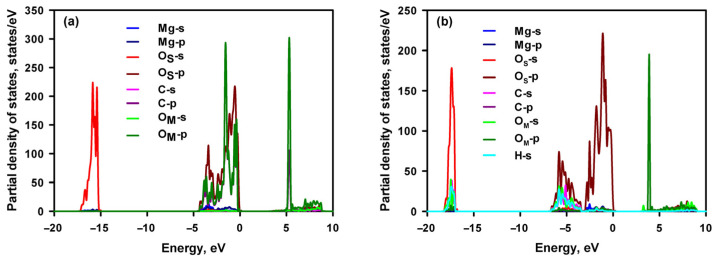
Projected density of states of MgO with adsorbed CO_2_ (a) and Mg(OH)_2_ with adsorbed CO_2_ (b), respectively. O_M_ and O_S_ refer to the O of CO_2_ and O of the adsorbent, respectively.

The optimised configurations for MgO and Mg(OH)_2_ with adsorbed CO_2_ and CH_4_ are shown in Figure 1. The attractive/repulsive interaction between molecules was not taken into account when estimating the adsorption energy in this simulation because only one adsorbate molecule was introduced to the surface. According to our preliminary research, the adsorption energy of the adsorbate molecule on the top of the lattice oxygen of MgO (Figure 2a) was the highest among the four potential adsorption sites, i.e., the two-fold bridge, the four-fold hollow, on the top of the oxygen anion, and on the top of the magnesium cation. However, among the three potential adsorption sites—the two-fold bridge, the four-fold hollow, and the top of the hydrogen cation—the adsorbate molecule’s adsorption energy was the highest on top of the four-fold hollow of Mg(OH)_2_. This is consistent with earlier findings [39,40].

Table 3 lists the adsorption properties for CH_4_ and CO_2_ on the MgO and Mg(OH)_2_ surface. MgO and Mg(OH)_2_ exhibit strong and weak adsorption to CO_2_, respectively. The adsorption energy difference is 0.525 eV. Contrarily, CH_4_ exhibits weak adsorption on both MgO and Mg(OH)_2_. The adsorption energy difference is 0.015 eV. This means that MgO has a strong attraction to CO_2_, while Mg(OH)_2_ has a mild one. An interwoven composite with alternate layers of MgO and Mg(OH)_2_ is anticipated in order to improve CO_2_ adsorption, which employs a mechanism similar to that of the Namib Desert beetle. Additionally, a composite comprised of MgO and Mg(OH)_2_ would have little impact on the adsorption of CH_4_ because of CH_4_’s modest affinity for both of these substances. This suggests that CO_2_ and CH_4_ in the biogas might be separated.

The distance from CO_2_ to the MgO surface is shown in Figure 1; it is 1.517, which is more than the C-O bond length of 1.43 mm [41]. This demonstrates that the adsorbed CO_2_ does not chemically react with MgO to form carbonate; therefore, the adsorption can be assumed to be strong physical adsorption. Since the distance between CO_2_ and the Mg(OH)_2_ surface is significantly longer than the length of the C–O bond, it is likely due to weak physical adsorption. CH_4_ is farther away from MgO and Mg(OH)_2_ surface than CO_2_. Perhaps the steric effect is involved here. This agrees with the average angle depicted in Figure 1. The average H–C–H angle of CH_4_ changes from 109.5° to 109.4° on the Mg(OH)_2_ surface and 109.0° on the MgO surface, respectively, indicating the stiffness of the CH_4_ molecule. On the other hand, the C–O–C angle of CO_2_ changes from 180.0° to 179.0° on the Mg(OH)_2_ surface and 133.6° on the MgO surface. This proves that MgO highly polarises the CO_2_ molecule. Table 3 shows that the CO_2_ molecule accepts a charge of 0.40, indicating that MgO donated 0.40 electrons to the CO_2_ molecule. The CO_2_ may become polarised as a result of MgO’s transfer of electrons to it. The fact that MgO and Mg(OH)_2_ both gave very few electrons to CH_4_ molecules points to a weak interaction with the surface. As a result, CH_4_ can be regarded as being weakly physically adsorbed by MgO and Mg(OH)_2_.

Figure 2 displays the projected density of states (PDOSs) of the atoms for CO_2_-adsorbed MgO and Mg(OH)_2_. The significant hybridisation between the O of CO_2_ and Mg of MgO, as well as between the C of CO_2_ and O of MgO, is observed in Figure 2a. The peaks of the PDOSs for the two atoms overlap between −5.0 and 0 eV. The strong hybridisation stabilises the CO_2_ molecule on the MgO surface. There is also hybridisation between the O of Mg(OH)_2_ and the C of CO_2_ since their PDOSs share some peaks. However, as can be seen in Figure 2b, the overlap is slight. Therefore, the weak hybridisation results in a low adsorption energy of CO_2_ on the Mg(OH)_2_ surface.

**Figure 3:**
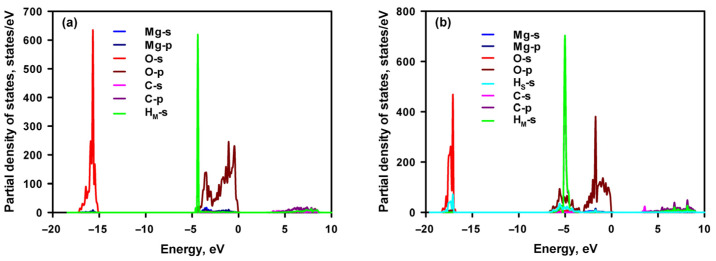
Projected density of states of MgO with adsorbed CH_4_ (a) and Mg(OH)_2_ with adsorbed CH_4_ (b), respectively. H_M_ and H_S_ refer to the H of CH_4_ and H of the adsorbent, respectively.

Figure 3 depicts the PDOSs of CH_4_-adsorbed MgO and Mg(OH)_2_. Because the PDOSs of MgO or Mg(OH)_2_ and CH_4_ do not have any common peaks, this indicates that no appreciable hybridisation occurs between the atoms of either MgO or Mg(OH)_2_ and the CH_4_ molecule. As a result, the CH_4_ molecule has poor adsorption on both the MgO and Mg(OH)_2_ surfaces.

In conclusion, the adsorption energies computed in Table 3 match the trends discovered by FactSage computations, providing more nuanced understandings: (1) CO_2_ exhibits stronger adsorption (−0.727 eV) than CH_4_ (−0.173 eV) on the MgO surface. MgO does absorb CH_4_, according to the surface model, contrary to Equation 4 in the bulk model. (2) The Mg(OH)_2_ surface exhibits the same trend (−0.202 eV for CO_2_ and −0.158 eV for CH_4_) as the bulk model. (3) In contrast to what is implied by the bulk model, CH_4_ forms a slightly stronger bond on MgO (−0.173 eV) than on Mg(OH)_2_ (−0.158 eV). (4) MgO exhibits a much stronger bond with CO_2_ (−0.727 eV) than Mg(OH)_2_ (−0.202 eV). According to these results, the interweaving of MgO and Mg(OH)_2_ structures may increase the effectiveness of selective CO_2_ capture relative to CH_4_.

### 3.3. Measurements of Selective CO_2_ Capture over CH_4_ in S1, S2, and S3 Systems

**Figure 4:**
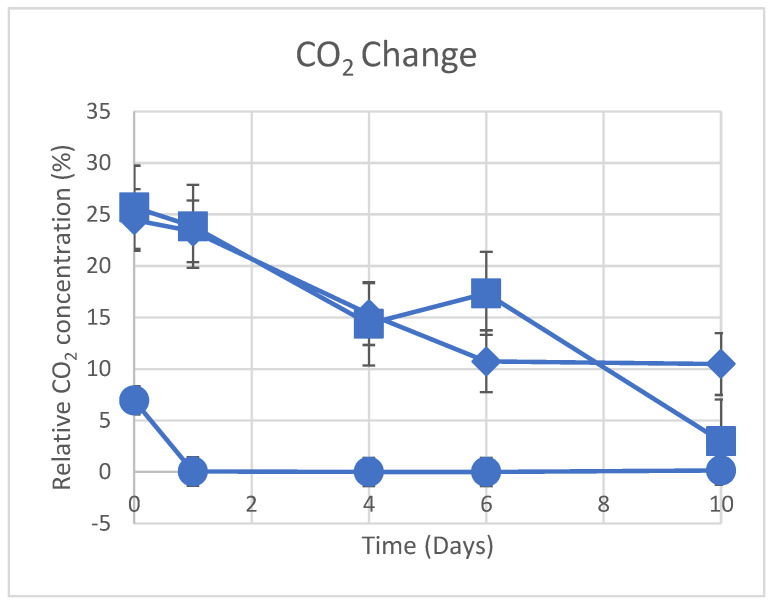
Variation in CO_2_ concentration with time during purification and under shaking at 150 rpm. S2 (●), S3 (■), and S1 (◆)**.**

**Figure 5:**
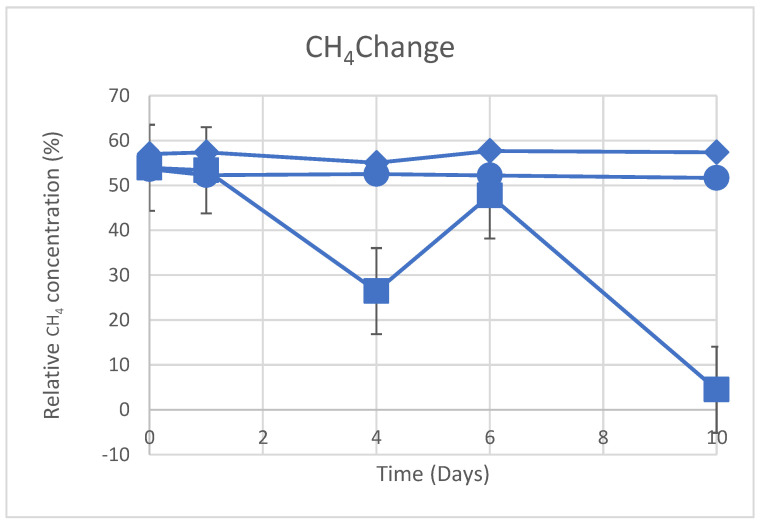
Variation in CH_4_ concentration with time during purification and under shaking at 150 rpm. S2 (●), S3 (■), and S1 (◆)**.**

The dynamic changes in CO_2_ concentration seen in photobioreactors are shown in Figure 4 using biological S1 and chemical S2/S3 approaches. Over a 10-day period at 150 rpm, S1 (photosynthetic bacteria) and S3 (MgO solid powder) consistently reduce CO_2_ concentration, while S2 completely eliminates CO_2_ on the first day. Figure 5 displays changes in the observed CH_4_ concentration over time in photobioreactors. S1 and S2 both show a negligible drop in CH_4_ during a 10-day period at 150 rpm. However, S3 (the introduction of MgO solid powder in the photobioreactor) causes the CH_4_ reduction to fluctuate, which is consistent with the results of our DFT simulation shown in Table 3 (−0.173 eV for CH_4_/MgO adsorption). The simulation indicates that the adsorption energy of CH_4_ on the MgO surface is stronger than that of CH_4_ on Mg(OH)_2_, providing an explanation for the observed fluctuations in CH_4_ concentration when MgO solid powder is solely added. The abundant MgO surface sites in S3 contribute to the varying CH_4_ concentration.

Additionally, the CO_2_-phobic (Mg(OH)_2_) and CO_2_-philic (MgO) model [25,34], which is modelled after the water collection system used by the Namib Desert beetle [24], is responsible for the quick elimination of CO_2_ through the use of S2, where MgO-Mg(OH)_2_ particles are used due to metastable chemical equilibrium, as in the current study, the solubility of MgO is 0.0086 g/100 mL at 30 °C. We did not look at the early phases of the reaction because the effectiveness of our suggested method for purifying methane was the focus of our work. Neshat et al. [2] only observed a slight reduction in CO_2_ levels after three days and a 10% decline after ten days when employing purple photosynthetic bacteria for CO_2_ fixation from biogas, a significantly slower process compared to our combined approach utilising photosynthetic bacteria and adsorption.

In contrast to adsorbents that are only utilised for methane purification, PNSB demonstrated the potential for concurrent nutrient recovery and biogas upgrading from anaerobic digested wastewater. The S2 method and S1 for waste water treatment and resource recovery can be coupled to expedite the CO_2_ separation from CH_4_ process.

## 4. Conclusions

This study presents a comprehensive analysis of methane purification in anaerobic fermentation bioreactors, with a particular emphasis on selective CO_2_ absorption. We systematically evaluated the efficacy of chemical adsorbents (S2 and S3) and photosynthetic bacteria (PNSB) for CO_2_ capture and methane purification, employing a combination of modelling and experimental techniques.

Our investigation initially demonstrated the selective CO_2_ over CH_4_ behaviour in MgO and Mg(OH)_2_ systems through bulk thermodynamic equilibrium modelling. Although it could not predict CH_4_ absorption on MgO, the surface DFT modelling results confidently predicted excellent CO_2_ selectivity for MgO-Mg(OH)_2_ composites, a prediction substantiated by on-site measurements.

PNSB (S1) exhibited commendable CO_2_ removal, achieving a 40% reduction over 10 days. In contrast, the S2 (MgO-Mg(OH)_2_ composite) showed remarkable speed, achieving complete CO_2_ removal within a single day while retaining 100% of the original CH_4_ content in the biogas. In contrast, S3 (solid MgO powder) was less effective, preserving only 5% of CH_4_ after a 10 h reaction. Consequently, S2 demonstrated an unparalleled CO_2_ removal speed, outperforming PNSB by a factor of 10.

Drawing from these results, we propose an innovative hybrid method that leverages the rapid CO_2_ removal capability of S2 and the superior nutrient recovery attributes of S1 PNSB. This approach holds the potential to revolutionise methane purification in anaerobic fermentation bioreactors, enhancing both efficiency and sustainability. Moreover, it can be seamlessly integrated into existing bioreactors with the addition of an adsorption module, making it highly practical.

## Figures and Tables

**Table 1:** Components of bacterial growth medium of 1 litre.

Component	Yeast Extract	CH_3_CH_2_COONa	NH_4_Cl	K_2_HPO_4_	NaCl	MgSO_4_•7H_2_O	Concentrated Trace Salt Solution *
amount	10 g	100 g	2 g	2 g	1 g	0.4 g	50 mL

* Concentrated trace salt solution was prepared by mixing 50 mL of de-ionized water with 1 g of FeCl_3_•6H_2_O, 2 g of CaCl_2_, 0.2 g of MnCl•4H_2_O, and 0.1 g of Na_2_MoO_4_•2H_2_O.

**Table 2:** Calculated equilibrium reactions of CO_2_ and CH_4_ absorption using MgO and Mg(OH)_2_.

Reactants		Products	
MgO + H_2_O 20.0 g 100.0 g	=>	H_2_O + Mg(OH)_2_ 91.1 g 28.9 g	(1)
MgO + CO_2_ 20.0 g 10.0 g	=>	MgO + MgCO_3_ 10.8 g 19.2 g	(2)
CO_2_ + H_2_O + Mg(OH)_2_ 10.0 g 91.1 g 28.9 g	=>	H_2_O + MgCO_3_ + Mg(OH)_2_ 95.1 g 19.2 g 15.7 g	(3)
MgO + CH_4_ 20.0 g 10.0 g	=>	MgO + CH_4_ 20.0 g 10.0 g	(4)
Mg(OH)_2_ + H_2_O + CH_4_ 28.9 g 91.1 g 10.0 g	=>	Mg(OH)_2_ + H_2_O + gas mixture (CH_4_ + H_2_O) 28.9 g 90.8 g 10.3 g (10.0 g + 0.3 g)	(5)

**Table 3:** Adsorption properties for CH_4_ and CO_2_ on MgO and Mg(OH)_2_.

Adsorbate	E_ad_ (eV)		Charge	
	MgO	Mg(OH)_2_	MgO	Mg(OH)_2_
CH_4_	−0.173	−0.158	0.02	0.00
CO_2_	−0.727	−0.202	0.40	0.01

## Data Availability

The datasets used and/or analysed during the current study are available from the corresponding author upon reasonable request.
